# Investigation of the Freezing Phenomenon in Vials Using an Infrared Camera

**DOI:** 10.3390/pharmaceutics13101664

**Published:** 2021-10-12

**Authors:** Maitê Harguindeguy, Lorenzo Stratta, Davide Fissore, Roberto Pisano

**Affiliations:** Department of Applied Science and Technology, Politecnico di Torino, 24 Corso Duca degli Abruzzi, 10129 Torino, Italy; maite.harguindeguy@polito.it (M.H.); lorenzo.stratta@polito.it (L.S.); roberto.pisano@polito.it (R.P.)

**Keywords:** freeze-drying, freezing, IR imaging, VISF, suspended vials, model

## Abstract

The freezing phenomenon has a dramatic impact on the quality of freeze-dried products. Several freezing models applied to solutions in vials have been proposed to predict the resulting product morphology and describe heat transfer mechanisms. However, there is a lack of detailed experimental observations of the freezing phenomenon in vials in the literature. Thus, the present work offers new experimental observations of the freezing phenomenon in vials by infrared (IR) thermography. IR imaging allowed each vial’s whole axial temperature profile to be collected during freezing, providing significant insights into the process. Spontaneous nucleation and vacuum-induced surface freezing (VISF), as a controlled nucleation technique, are investigated. Batches having vials in direct contact with the shelf (exchanging heat mainly through conduction) as well as suspended (exchanging heat mainly through natural convection and radiation) were tested. The study used three solutions: sucrose 5%, mannitol 5%, and dextran 10%. SEM images coupled with an automated image segmentation technique were also performed to examine possible correlations between the freezing observations and the resulting pore size distributions. IR thermography was found to be a promising tool for experimentally predicting the resulting product morphology in-line.

## 1. Introduction

The pharmaceutical industry has undergone a deep renovation during the past decade, shifting its research and development efforts from chemically synthesised drugs to biopharmaceuticals [1]. The several benefits of biopharmaceuticals can explain this trend shift. Their benefits include highly effective and potent action, few side effects, and the potential to cure previously untreatable diseases [2]. Moreover, the pandemic caused by COVID-19 highlighted the necessity to have reliable ways to stabilise and store therapeutic liquid products, e.g., vaccines, for long times. These kinds of drug products are, in fact, often unstable in solutions and lose their activity when subjected to the high temperatures used in conventional drying [2]. Freeze-drying is a good fit for these drugs because it is a dehydration technique performed at low temperatures, increasing the product’s shelf-life while maintaining its biological activity.

Freeze drying can be divided into three steps: freezing, primary, and secondary drying [3]. During freezing, the solution containing the drug is frozen and cooled to temperatures close to 223 K. Then, heat is supplied to the product during primary drying, and vacuum is applied; the pressure is lowered to few Pascals (Pa), and the solvent—usually water—is removed through sublimation. Finally, during secondary drying, the temperature of the product is increased to remove the remaining adsorbed solvent on the solid product matrix.

The freezing step has been recently studied in detail as it was proven to substantially influence the product structure [4,5,6] and the drug residual activity [7,8,9,10]. When water is cooled below its equilibrium temperature, it can remain in the liquid state for a relatively long time before the occurrence of the phase transition into solid ice. This phenomenon is called supercooling and represents a metastable state for water. Even though ice formation is thermodynamically favoured, being below its equilibrium temperature, the system must surpass an energy barrier to form the first ice nuclei. This energy barrier depends strongly on the temperature and defines a critical nuclei dimension. Creating an ice-water interface with nuclei below this critical value would require more energy than the one released as latent heat of solidification by the nucleus. As the supercooling degree increases, the critical diameter decreases rapidly, increasing the probability of the appearance in the solution of a stable nucleus [11,12,13,14]. After forming the first nuclei, also called nucleation, part of the solution freezes instantly, releasing energy and increasing its temperature to its equilibrium temperature [15]. The number and dimension of the ice crystals, which are the casts of the pores in the freeze-dried cake, depend on the nucleation temperature. At low nucleation temperatures, numerous small nuclei form and grow, while at higher temperatures, fewer crystals but of larger dimensions are obtained [5].

Moreover, the distribution of the pores size distribution correlates with the resistance to the mass transport of the dried cake ($R_{p}$) in the primary drying stage when the water vapour moves from the interface between the frozen and the dried product to the chamber through the already dried product. In turn, the $R_{p}$ affects the primary drying phase in terms of duration and maximum temperature reached by the product [16].

Spontaneous nucleation usually occurs at low temperatures with large vial-to-vial variability. This condition leads to tremendous resistances to mass transport and large variability of the drying time inside a batch [17]. The most recent controlled nucleation techniques aim to overcome these problems by directly and precisely controlling the nucleation temperature and, therefore, the pore-size distribution. Among these techniques are annealing [18,19,20], pre-cooled shelves [4], ice-fog, electric field-induced nucleation [21], and vacuum-induced surface freezing (VISF) [22,23,24,25,26], amongst others. VISF is interesting since it does not require changes in the freeze-drying equipment to be implemented. All it needs is an isolation valve between the condenser and drying chamber and manual or semi-automatic control over the vacuum pump and condenser operation.

Freezing is a highly stochastic phenomenon, and much of the product variability in a batch comes from having vials nucleating at different nucleation temperatures. Thus, a better understanding of the freezing phenomena and its implications on product morphology is essential. Controlled freezing techniques have recently gained popularity in the freeze-drying field to increase batch homogeneity and improve product quality control [17].

Many papers deal with the definition of mathematical models to describe the freezing of pharmaceutical solutions and predict the ice crystals’ size distribution. Many of these models are empirical, like the one from Nakagawa et al. [27], while some are mechanistic, like the one proposed by Arsiccio et al. [28]. Recently, Colucci et al. presented a mechanistic approach based on the universal quasichemical model, describing nucleation and crystal growth using a one-dimensional population balance [29]. However, up to some extent, all these models are based on heat and mass transfer models themselves, not on direct experimental observation.

The use of an infrared camera to monitor freeze-drying processes was first proposed by Emteborg et al. [30]. In that study, the camera was placed on the top of the drying chamber, monitoring only the temperature at the top of the product. The same approach was used by Gonçalves et al. [31] to monitor the primary drying stage. Using an infrared sensor to monitor the axial vial profile was proposed by Van Bockstal et al. [32]. However, vials were frozen using the spin-freezing technique, and an IR heater assisted primary drying. This setup resulted in a very particular cake structure and temperature profiles. Lietta et al. [33] proposed monitoring a freeze-drying process using an IR camera inside the drying chamber, monitoring the whole axial temperature profile. Later, Colucci et al. attempted to monitor the freezing step revealing that thermal gradients could be observed using IR thermography [34].

This work shows new experimental observations of the freezing phenomenon in vials using an IR camera, comparing the different aspects of freezing. In particular, the effects of spontaneous nucleation versus induced nucleation using VISF are investigated. Then, the impact of the various heat transfer mechanisms occurring during freezing in the case of two different vial loading configurations: vials placed in direct contact with the temperature-controlled shelves and those suspended [35]. In the first case, heat is transferred mainly by conduction between the shelf and the vial bottom, and the thermal gradients can be approximated as unidirectional. In the latter, natural convection is predominant, and heat is transferred through the bottom and the side of the vials almost uniformly. The primary effect of this change is that, in the suspended configuration, freezing cannot be considered unidirectional anymore. Thus, the experimental observation of the freezing phenomenon is more challenging while mathematical models fail to predict the crystal size distribution. These considerations were evaluated using three different pharmaceutical formulations containing amorphous and crystallisable excipients and amorphous solids with different glass transition temperatures.

Our work brings new insights into the freezing phenomenon, such as the experimental temperature profiles, heat transfer insights, and their impact on the resulting dried cake structure. These new insights will be thoroughly discussed in this paper. We expect that the observations here presented may also be helpful in the development of future models or adjustments to the current ones, making model predictions more reliable.

## 2. Materials and Methods

### 2.1. Formulations and Experimental Apparatus

The solutions were prepared with an excipient concentration of 5% *w*/*w* for both sucrose and mannitol (Sigma Aldrich, Steinheim, Germany) and 10% *w*/*w* for dextran 40 Kd (PanReac AppliChem, Chicago, IL, USA). These solutes were dissolved in water for injection (WFI) (Fresenius Kabi, Verona, Italy) and filtered with 0.22 μm PVDF sterile filters (Merk Millipore, Cork, Ireland). Each 4R vial (Nuova Ompi glass division, Stevanato Group, Piombino Dese, Italy) was filled with 1 mL of solution, resulting in a 10 mm cake height and pre-stoppered with silicon stoppers (West Pharmaceutical Service, Milano, Italy).

All the experiments were conducted in a LyoBeta 25 (Telstar, Terrassa, Spain) freeze dryer. The freeze dryer is equipped with a capacitance manometer (Baratron type 626A, MKS Instruments, Andover, MA, USA) and thermal conductivity manometer (Pirani type PSG-101-S, Inficon, Switzerland). The ratio of the two signals was used to detect the endpoint of primary drying [36,37].

One vial per batch was used to monitor the temperature with a T-type miniature thermocouple (Tersid, Milano, Italy) as a control vial. This measurement was done to ensure an extra temperature monitoring control in real-time as a reference. Still, the thermocouple data were not post-processed, and it is not presented here since the IR data was richer than the thermocouple one. All the temperature profiles were monitored using an infrared (IR) sensor system (IMC Service S.r.l., Mascalucia, Italy), the same sensor used and described in some previous works [34,38]. This system includes a built-in thermal camera (FLIR Systems model A35; FLIR Systems Inc., Wilsonville, OR, USA), a processing board, and a Wi-Fi antenna for wireless data transfer.

**Figure A1:**
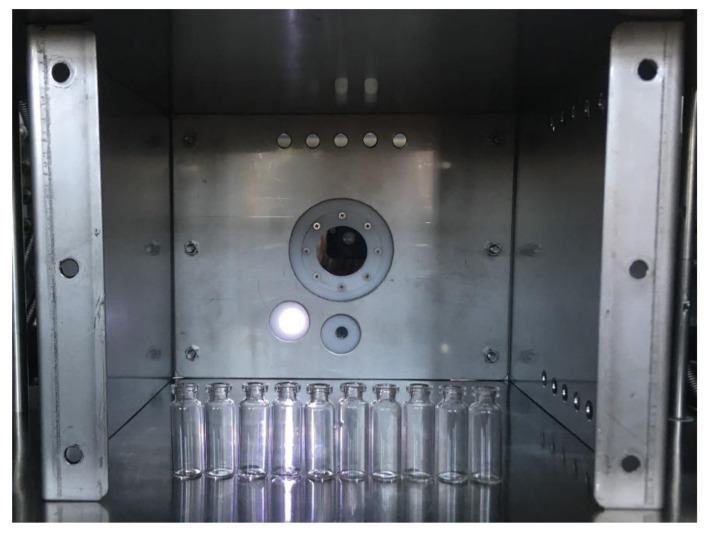
Picture of the box used with the IR sensor behind it and 10 vials placed ~25 cm from the sensor, as set for the experiments. The front wall of the box was removed for the picture.

In all the runs, the vials were surrounded by a customised stainless steel box (260 × 280 × 205 mm^3^ as width, depth, and height) to mitigate the contribution of the radiation coming from the non-thermally controlled chamber walls of the freeze dryer. The metal box was carefully designed with specific holes to observe the vials with the IR sensor system from outside at an approximate distance of 25 cm and to guarantee vacuum conditions during primary and secondary drying. Moreover, the box was placed in direct contact with two adjacent shelves to ensure thermal uniformity between the shelves and the walls of the box. In this way, the box walls had a temperature profile at least 10 K lower than the chamber walls for most of the process. The IR sensor was placed between the back wall of the freeze dryer and the metal box. Figure A1 in the annex depicts the system setup.

### 2.2. Freeze-Drying Protocols

**Figure 1:**
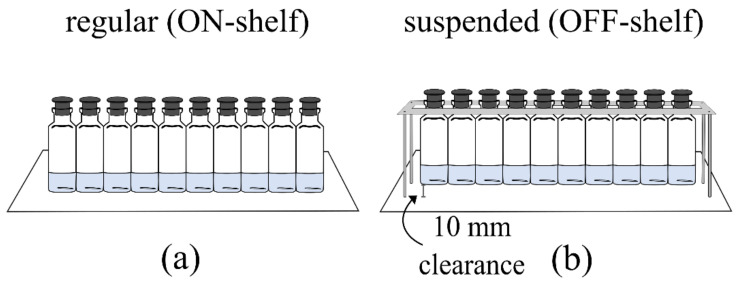
Experimental setup representations (not to scale): (**a**) the ON-shelf and (**b**) OFF-shelf vial configurations used.

For all the experiments, a total of 10 vials were used. Two different configurations were used to test the effect of varying heat transfer scenarios, as described in Figure 1. In the first one, the vials were directly in contact with the shelf; thus, the heat was mainly transferred by direct conduction between the shelf and the vial bottom. This configuration will be called ON-shelf throughout the paper. In the second one, the vials were suspended with two Plexiglass tracks held by screws, as presented by Capozzi et al. [35], having a 10 mm ± 1 mm clearance to the shelf. In this way, the heat was transferred only by natural convection and radiation from the temperature-controlled shelves and the box walls. This latter configuration will be called OFF-shelf throughout the paper.

In all the controlled nucleation experiments, the vials were first equilibrated at the selected nucleation temperature (*T*_n_) for ~1 h, and nucleation was induced using VISF [25,26]. The chamber pressure was reduced as fast as possible to a product-specific value (~1 to 2 mbar) and held until nucleation occurred in all the vials. Then, the chamber was restored to atmospheric pressure using a stream of nitrogen gas. During VISF, fast cooling of the upper layer of the solution is triggered by the evaporation caused by the vacuum. If the procedure is performed fast enough, the surface of the solution cools so much that nucleation is firstly confined to the most superficial layer of the liquid and then rapidly extends, within a few seconds, to the entire volume of the supercooled solution, which is at the desired $T_{n}$. Applying VISF in a process guarantees a uniform nucleation temperature throughout the whole batch of vials [23]. The influence of the nucleation temperature on the average ice crystal size was tested upon two values of $T_{n}$, one close to the equilibrium temperature (271 K) and the second on the limit of observing spontaneous nucleation for the solutions used (263 K). The shelf temperature was set to obtain similar values of $T_{n}$ for the two loading configurations tested (Figure 1), i.e., 268 K ($T_{n}$ = 271 K) and 258 K ($T_{n}$ = 263 K) for the ON-shelf vials and 262 K ($T_{n}$ = 271 K) and 248 K ($T_{n}$ = 263 K) for the OFF-shelf vials. After nucleation, the temperature of the shelf was maintained at the holding temperature $T_{h}$ for 1 h to ensure complete freezing. The chosen $T_{h}$ depended on the loading configuration and was the same one used to obtain a $T_{n}$ of 263 K in all the experiments, i.e., 258 K for the ON-shelf vials and 248 K for the OFF-shelf ones. In this manner, the degrees of freedom of the freezing operating conditions are reduced. After the holding time, the temperature of the shelf was lowered to 223 K at a rate of 0.5 K/min and maintained for 2 h at 223 K. For the experiments involving spontaneous nucleation, the product was held at the specified $T_{n}$, i.e., the one to obtain a 263 K product temperature until nucleation occurred in all the vials. The nucleation of all batch vials was assessed by visual inspection. After that, the shelf temperature was lowered to 223 K at a rate of 0.5 K/min and maintained at that value for 2 h.

The drying protocol was the same for all the experiments after freezing. The chamber pressure was lowered to 5 Pa, and the shelf temperature was increased from 223 K to 253 K as fast as possible (~1 h). The latest conditions were then maintained for 15 h to complete primary drying fully. The shelf temperature was then raised from 253 K to 293 K in a 4-h-ramp and kept at 293 K for two hours to complete secondary drying. At the end of the process, the vacuum was broken with a nitrogen stream. Then, the vials were stoppered, sealed with aluminium caps, and stored in a freezer at 253 K for further evaluation.

### 2.3. IR Data Acquisition and Processing

**Figure 2:**
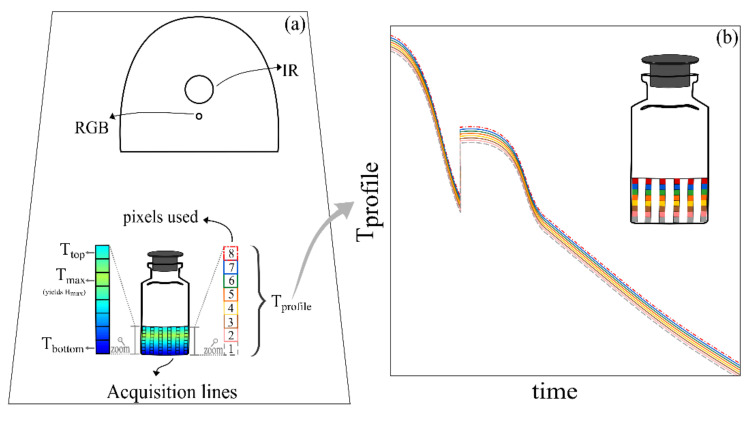
Data acquisition scheme. (**a**) Acquisition lines and pixel resolution used to obtain the IR-based thermal measurements. The colours and line types used as described in this figure by “pixels used” are consistently used to represent data acquired from those pixel positions throughout the paper when applicable. The colour gradient in the vial represents the thermal gradient detected by the camera and expresses the detection and definition of the $T_{max}$ and $H_{max}$. (**b**) a representation of the $T_{profile}$ that would be obtained from a vial with an arbitrary consistent thermal gradient, increasing from bottom to top.

Thermal data are acquired by setting the acquisition lines during post-processing of the acquired IR images. The bottom and top pixels of the desired acquisition lines are defined, and all pixels vertically between these two points are called an acquisition line as schematically depicted in Figure 2. The same IR camera used in this study was previously applied to monitor the freezing step in vials subjected to spontaneous nucleation [34]. However, the data acquisition algorithm used had some fundamental changes. The previous study used three acquisition lines per vial to obtain the vial’s average profiles. These acquisition lines were first filtered using a Savitzky–Golay filter built-in MATLAB function. Then, the three acquisition lines per vial were averaged to give the vial’s average axial temperature profile and filtered with the same tool mentioned above. Then, based on the average profile, the temperature values ($T_{min}$ and $T_{max}$) and position ($H_{min}$ and $H_{max}$) of the pixel with the minimum and maximum temperature were extracted through an analysis of the first and second derivate of the profile [39]. In this present study, more acquisition lines per vial are used (six), and all noise filtering tools were removed. Additionally, the data acquisition order and the averaging used were changed. In the results ahead, the value ($T_{min}$ and $T_{max}$) and position ($H_{min}$ and $H_{max}$) of the minimum and maximum pixel from each of the six acquisition lines was recorded using the *min* and *max* MATLAB built-in tools. Then, the average temperature value and position from the six acquisition lines were computed to obtain the vial’s average profile values. Besides simplifying the data processing algorithm, these changes allowed a much more detailed observation of the freezing phenomenon using the same hardware.

The custom acquisition software used was developed on MATLAB (version 9.7.0 R1019b, The MathWorks, Portola Valley, CA, USA), based on the work presented by Harguindeguy & Fissore (2020) [38]. As portrayed in Figure 2, each vial had six vertical data acquisition lines equally spaced across the vial. Each of these lines had 8 pixels, covering the whole cake height. The pixel presenting the maximum axial temperature ($T_{max}$) in each line was tracked and its distance from the vial bottom was called $H_{max}$ in this paper. The $T_{max}$ position and temperature reading in each group of the six vertical acquisition lines was averaged to determine the vial $T_{max}$. Then, the average temperature and position of the $T_{max}$ was averaged throughout all the vials to determine the batch $T_{max}$ mean profiles.

The software uses a constant emissivity value throughout the whole process (ε = 0.91). However, small changes in the vial emissivity happen during a freeze-drying cycle. An emissivity correction method was applied when processing the data as previously described [38].

### 2.4. SEM Analysis

The dimension of the pores was analysed using a Desktop SEM Phenom XL (Phenom-World B.V., Eindhoven, Netherlands) at an accelerating voltage of 15 kV. The samples were extracted from the vials and cut vertically. The slice was then fixed on an aluminium circular stub and metallized with platinum using a sputter coater (Balzer AG, type 120B, Balzers, Liechtenstein). Three SEM pictures of the product were taken on the vertical axis at the top, centre, and bottom using magnifications from 210× to 270×.

The pore size distribution was then obtained with the Multivariate Image Analysis (MIA) technique described by Colucci et al. [34]. The SEM images obtained had an image resolution of 1024 × 1088 pixels. Multivariate image analysis (MIA) techniques [40] were used to segment the regions of the images corresponding to the single pores and measure the distribution of the axial pores inside the product [34]. The brightness of the SEM images was first equalized to reduce the charging effect, using a moving average filter [41]. For each image, a “Bharati matrix” [3] was created. This is a data matrix that accounts for the intensities of the single pixels and the textural relation between the intensity of adjacent pixels. A principal component analysis (PCA) model [42,43] was extracted from the obtained data structure. A moving window was used such that each variable corresponded to the intensity of one of the pixels in the moving mask. The pores were segmented, selecting all the pixels having a score higher than a given limit manually tuned. This procedure resulted in a dummy image where the segmented pores were highlighted as areas of ones while all the remaining pixels were marked as zeros.

A second filter was used to segment the lighter regions of the picture and those distinguished by remarkable gradients of pixel intensity. Then, the Canny algorithm for edge detection [44] was applied to this aim. Finally, all the areas lower than 50 or greater than 1000 pixels were removed using a dimensional filter. The number of pores was counted, the perimeter and area of each pore were computed using the *regionprop* function [45,46]. The 25th and 75th percentiles were calculated from the pore diameters histogram obtained from every image.

### 2.5. Statistical Analysis

The pore size distribution within the freeze-dried samples was previously found to follow a beta distribution with a = 1.2 and b = 15 [29]. This assumption was tested using a Q-Q plot (presented in Figure A5 and Figure A6 in the Appendix A) for all performed tests. Each of the three evaluated cake levels per sample had at least three SEM pictures taken. A couple of thousands of pores, according to the sample, were considered in the statistical analysis using the MIA tool based on the data extracted from these pictures. Based on the pore size data, a variance and interquartile range analysis were done to compare the statistical dispersion of the pore size distribution. Variance is a measure of dispersion, meaning how far a set of numbers is spread out from their average value. The interquartile range finds where the middle half of the data values are; it evaluates where the bulk of the values lie. That is why it is preferred over many other measures of spread when reporting spread data, such as pore size distributions. The interquartile range was calculated by subtracting the first quartile from the third (Q3–Q1). All calculations were done in MATLAB (version 9.7.0 R1019b, The MathWorks, Portola Valley, CA, USA).

## 3. Results

### 3.1. Freezing Profiles: Spontaneous vs. Controlled Nucleation

**Figure 3:**
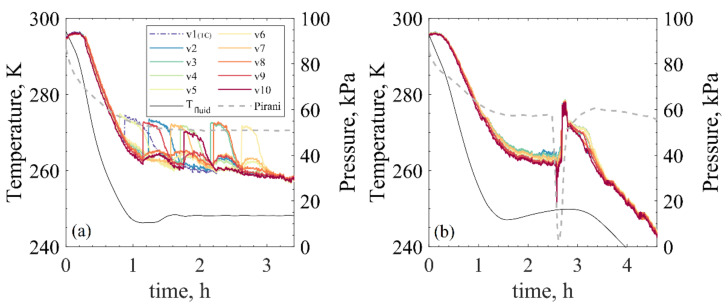
$T_{bottom}\;$freezing profiles of the spontaneous nucleation (**a**) and VISF at 263 K (**b**) in the case of OFF shelf vials and 5% sucrose.

First, the differences observed between spontaneous and controlled freezing should be stressed. In Figure 3, the bottom temperature profiles of all vials using the OFF-shelf configuration are presented, together with the operating conditions of shelf temperature and chamber pressure used. The OFF-shelf tests were chosen for this study because they presented detailed profiles. The ON-shelf spontaneous tests resulted in spread nucleation times for each vial, making these tests’ timescale less informative. From these figures, the first trivial observation is the stochastic nature of freezing when uncontrolled freezing is used instead of when controlled freezing is applied. Additionally, some humps on the temperature profiles (approx. 3–4 K) are observed for the spontaneous nucleation test caused by the nucleation energy released in neighbouring vials.

**Figure 4:**
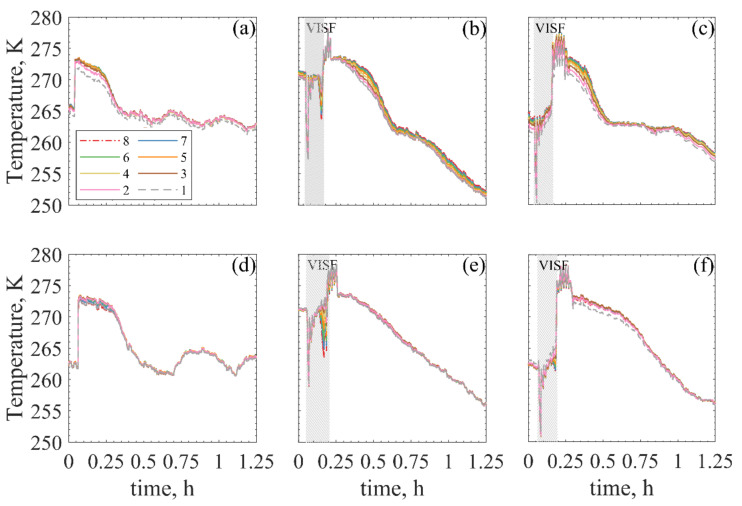
Axial temperature profiles during freezing of 5% sucrose. The top graphs (**a**–**c**) are ON-shelf, while bottom graphs (**d**–**f**) are OFF-shelf. (**a**,**d**) Spontaneous nucleation, (**b**,**e**) VISF 271 K, (**c**,**f**) VISF 263 K.

The axial temperature profiles observed in spontaneous and controlled nucleation of 5% sucrose using both the ON- and OFF-shelf loading configurations are shown in Figure 4. For the sake of brevity, the profiles observed for mannitol 5% and dextran 10% were not presented, but they present the same characteristics as the ones presented for sucrose 5%. Each line represents the temperature profile for a given pixel height and consequently, a cake height. The changes in the magnitude of the vertical temperature gradients within the vials can be monitored using these profiles. Therefore, changes in the temperature gradient direction, e.g., the coldest point of the vial’s axial temperature profile moving from the bottom to the top of the cake, can be observed if present.

In practice, water in solutions never freezes completely. After nucleation, pure ice forms, and the solution cryo-concentrates. Once the system reaches a specific concentration characteristic of each excipient, the cryo-concentrated solution remains in a supercooled single-phase amorphous state. Water molecules get trapped into the excipient solidified matrix and cannot diffuse and crystallise further [47,48]. For this reason, freezing is usually regarded as complete when the solution reaches the eutectic point ($T_{eu}$) for crystalline solutes or the glass transition temperature (${T_{g}}^{\prime }$) for amorphous ones. The ${T_{g}}^{\prime }$ for 5% sucrose is 241 K, 264 K for 10% dextran, while the $T_{eu}$ for 5% mannitol is 251.7 K [49,50]. However, in this study, the interval between nucleation and the product reaching nearly 263 K was the only one closely examined. During this interval, temperature gradients can be observed in the vial. However, after reaching around 263 K, for all tests, these gradients seem less evident. The release of the latent heat of solidification is less and less pronounced, and the product temperature tends to become more homogeneous. Moreover, the IR sensor may lack the sensitivity to detect any temperature gradients after that moment accurately. Thus, freezing is presented and discussed in-depth, only covering the interval as mentioned above. Following this definition, freezing takes approximately 20 min for ON-shelf vials and approximately 45 min for OFF-shelf ones.

**Figure A2:**
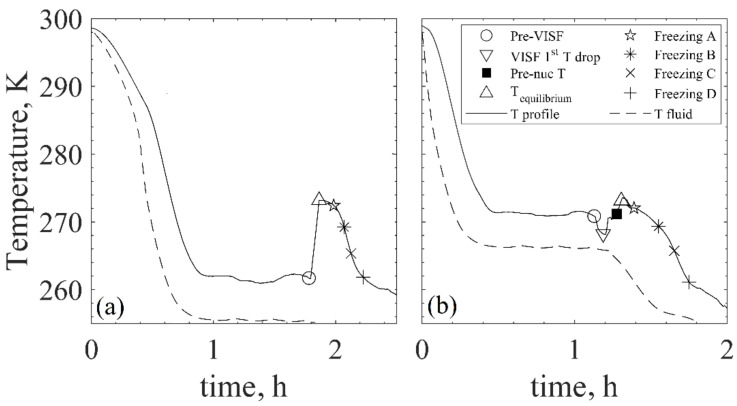
Representation of the points of interest during freezing for spontaneous nucleation (**a**) and VISF tests (**b**) that are used in Figure A3.

**Figure A3:**
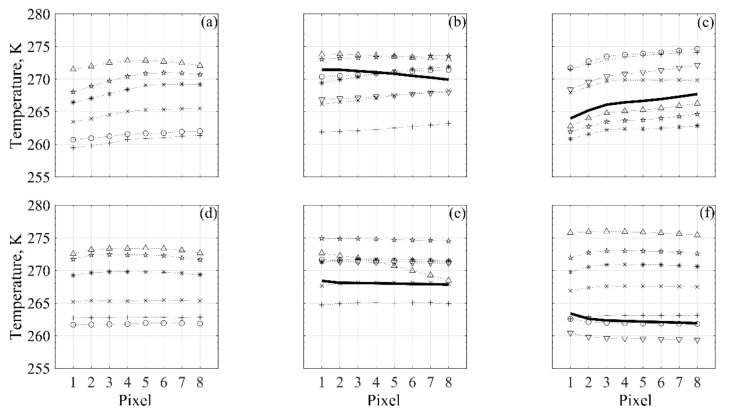
Vertical temperature gradients and possible profile inversions for sucrose 5%. Top graphs (**a**–**c**) are ON-shelf, while bottom graphs (**d**–**f**) are OFF-shelf. (**a**,**d**) Spontaneous nucleation, (**b**,**e**) VISF 271 K, (**c**,**f**) VISF 263 K. Each line represents the temperature gradient in the points of interest, represented by different symbols as shown in Figure A2.

As previously discussed, ice nucleation and freezing temperature directly affect the size distribution of the ice crystals formed. If a product has large temperature gradients, differences in the ice crystal size distributions may be observed. Since VISF cools down the top surface of the solution, it seemed interesting to investigate if this surface cooling could affect the product’s temperature gradient and if that could affect intra-vial homogeneity. A change in the temperature gradient direction was observed sometimes, and further exploratory information is needed. Hence, Table 1 lists whether the temperature profiles extracted from the bottom pixels were the lowest or highest temperatures observed for all tests. This table is aimed to help better analyse if there was an inversion on the vertical temperature gradients for any tested conditions and if this was consistent. Additionally, an alternative graph representation of the gradient profiles is represented in Figure A2 and Figure A3 in the Appendix A to better visualise the data.

In Table 1, the OFF-shelf tests were given an “Unclear” label regarding the temperature inversion. Observing the graphs in Figure 4c–e, the bottom temperature is the warmest before nucleation and the lowest after nucleation. However, this is also observed for the spontaneous nucleation test. Thus, the inversion cannot be necessarily attributed to the VISF application.

### 3.2. Freezing Front Temperature and Position

**Figure 5:**
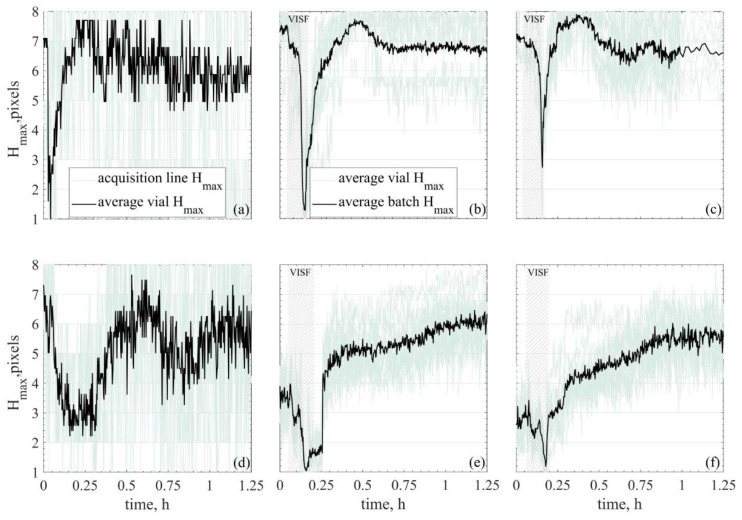
$T_{max}$ axial position ($H_{max}$) during the freezing interval of 5% sucrose. Top graphs (**a**–**c**) are ON-shelf, while bottom graphs (**d**–**f**) are OFF-shelf. For the spontaneous nucleation (**a**,**d**), $H_{max}$ is the average profile obtained from the six acquisition lines of a single vial (shadows plotted in light green). For VISF (**b**,**c**,**e,f**), $H_{max}$ is the batch average calculated from the average vial profiles of all ten vials (shadows plotted in light green). Graphs (**b**,**e**) are VISF at 271 K, (**c**,**f**) VISF at 263 K.

From profiling the whole axial temperature, the position of the $T_{max}$ was tracked and is plotted in Figure 5. Figure 5 shows the $H_{max}$ profile for sucrose 5% solutions. The profiles observed for mannitol 5% and dextran 10% followed the same trends and thus, were not presented. Because freezing is an exothermic phenomenon, if the heat exchange follows a vertical gradient, i.e., in the ON-shelf experiments, the freezing front position could be inferred by tracking the maximum temperature position within the product cake height. Once this profile is obtained, an evaluation can be done to determine whether this may be representative of the freezing front profile or not. In practical terms, an $H_{max}$ profile moving from bottom to top was observed when tracking the maximum temperature position, which agrees with what is expected from the freezing front. Still, since these are new observations, the documented profiles must be carefully evaluated to determine if the approximation $H_{max}\;profile≅freezing\;front\;profile$ is valid. This approximation is discussed in detail in Section 4.2.

**Figure 6:**
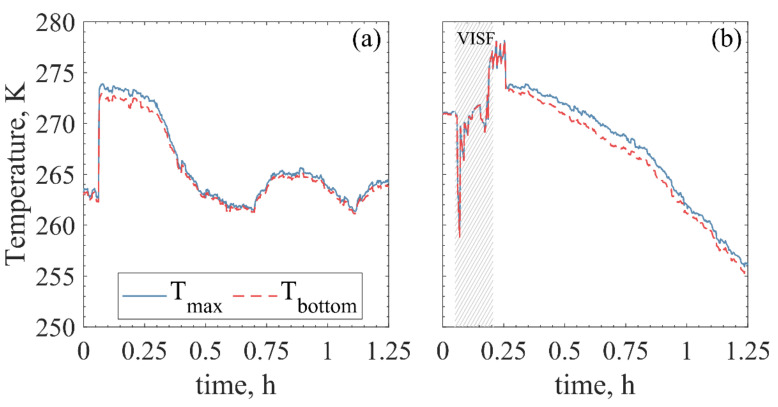
$T_{max}\;$and $T_{bottom}$ during spontaneous nucleation (**a**) and VISF (**b**) at 271 K. (These are the same tests represented in Figure 4d,e.

If the position of the freezing front can be inferred, the same can be done for the temperature gradient of the frozen layer. For uncontrolled nucleated ON-shelf vials, the $T_{min}$ will be the $T_{bottom}$ after nucleation. The $T_{top}$ will be, in fact, the $T_{max}$ for most of the freezing. However, that is not necessarily the case during the first minutes of freezing (~6–12 min) after controlled nucleation takes place. This time scale is in agreement with what was previously modelled for the freezing front of 10% mannitol using a $T_{n}$ of 266 K with a cake depth of 10 mm as well [27]. In the present experiments, in this time interval, the $T_{max}$ seemed to move upwards, from bottom to top for the ON-shelf vials. A much less evident profile was observed for the OFF-shelf vials, where the maximum temperature seemed to fluctuate around the central height of the vial’s pixels. Nonetheless, for the ON-shelf vials, the $T_{max}$ position and temperature could give information on the freezing front profile. In this case, the temperature gradient between the freezing front and the vial bottom could be inferred, as shown in Figure 6.

### 3.3. Product Cake Morphology

In 1991, Bald et al. proposed crystal size to be proportional to the rate of temperature change in the system [51]. Following this, the velocity of a solidification (freezing) front and the temperature gradient in the frozen product were defined as driving factors determining the ice crystal size [22,27]. Published works using ice crystal prediction models based on these concepts assumed a one-dimensional freezing front evolution, moving from bottom to top [22,27,52,53]. Later, a mechanistic model to predict ice crystal size distribution was proposed by Arsiccio et al., also based on this one-dimensional assumption for the freezing front evolution [28].

Arsiccio et al. reported that VISF allowed achieving narrower nucleation temperatures range reaching a more uniform product morphology than when spontaneous nucleation was used. The slight heterogeneity observed on the product when using VISF was due to the broader temperature gradient at the top and bottom of the sample. These gradients happen because of proximity to the shelf or the vial headspace [23]. The most uniform structure was found for 263 K in that study. Later, Capozzi et al. reported a more uniform cake structure for suspended vials than non-suspended ones using shelf ramp freezing [35]. Thus, in this study, we investigated what was left: if differences could be observed between ON and OFF shelf vials using VISF, a technique that already reduces the vial temperature gradient during nucleation.

Analysing the experimental observations and concepts from one-dimensional freezing models, some expectations regarding the resulting cake structures emerge. The OFF-shelf vials, which present narrower overall temperature gradients, should result in more homogeneous cakes. On the other hand, the ON-shelf vials with broader vertical temperature gradients should have a less homogeneous cake structure.

**Figure 7:**
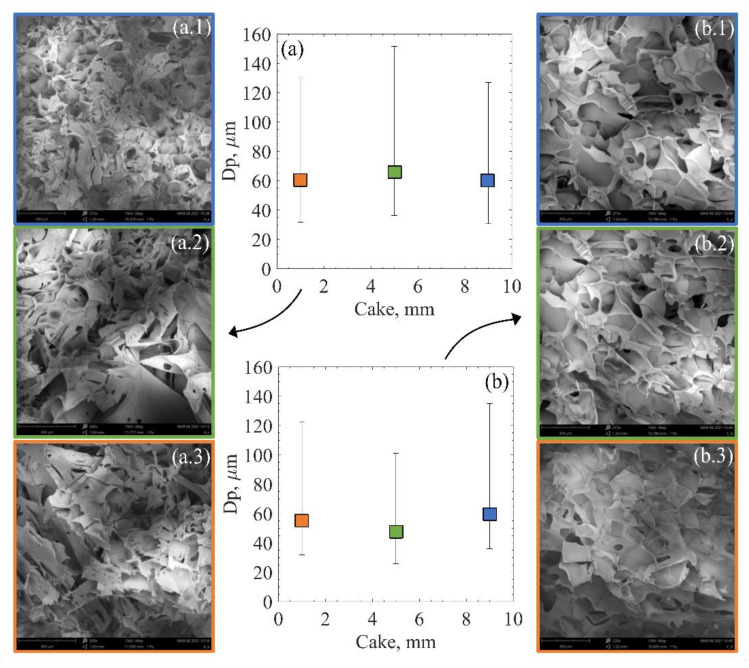
Average pore sizes for the resulting mannitol 5% cake after VISF tests at 271 K for ON-shelf (**a**) and OFF-shelf vials (**b**). The numbers after the test type letters (**a**,**b**) represent the cake section: (1) top, (2), middle and (3) bottom for the corresponding SEM images. The variability is expressed as the 3rd (**top**) and 1st (**bottom**) quartiles, as previously done for pore size data [28,35].

VISF was found to produce product matrices with larger pores than those obtained using spontaneous nucleation [35]. The OFF-shelf vials using uncontrolled nucleation were found to render cakes with larger pores and higher batch homogeneity than the ON-shelf vials. Another pertinent question to be verified was whether VISF coupled with the suspended vial configuration could produce products with a more homogeneous cake structure besides batch homogeneity. Figure 7 shows the SEM image results for the resulting cake structure of 5% mannitol solutions.

**Figure 8:**
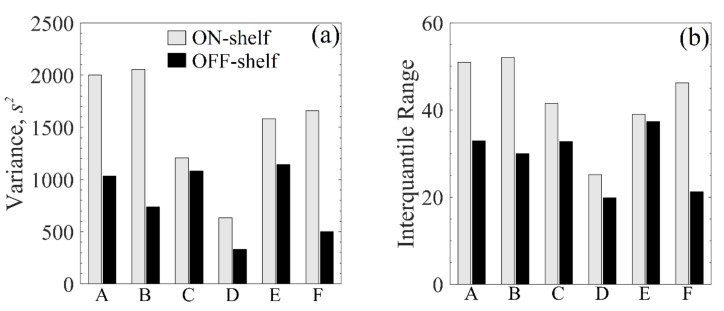
Variance (**a**) and Interquartile range (**b**) of the resulting pore size distribution for all VISF tests. In both bar plots, A, B and C are tests at 271 K while D, E and F are tests at 263 K. The solutions used are 5% sucrose (A,D), 5% mannitol (B,E) and 10% dextran (C,F).

Figure 8 shows different statistical parameters to measure the statistical dispersion of samples in a group: the variance and the interquartile (Q3–Q1) range. As seen from this figure, although the differences are not very pronounced, the OFF-shelf vials have consistently lower variability indicators, further discussed in Section 4.3.

## 4. Discussion

### 4.1. Freezing Profiles: Spontaneous vs. Controlled

In the uncontrolled nucleation graph of Figure 3, humps in the temperature profile were observed. Those humps come from the energy released during the nucleation of neighbouring vials since all vials were in contact during the tests. This energy release may slightly increase vial temperature or simply affect the IR measured values. After nucleation, warmer temperature traces may remain in the vial glass until equilibrium is reached between the vial wall and the product. When VISF was applied, since all vials nucleated around the same time (~100 s range), these interferences were not observed. However, a very pronounced initial peak on the temperature profile was observed just as nucleation occurred in all vials. This peak in the temperature readings was also attributed to this energy release during nucleation. Both Figure 3 and Figure 4 show that the nucleation heat-release hump was present for the VISF batches. This peak was not observed when spontaneous nucleation was used because the neighbouring vials could absorb this released energy easily.

From the axial temperature profiles shown in Figure 4, differences in the vertical temperature gradient could be observed between the different case studies. As expected, the ON-shelf vials had a broader vertical axial temperature gradient than the OFF-shelf vials.

For the ON-shelf vials, the bottom temperature, as expected, was the lowest. Then, VISF is applied, and an inversion is observed for the tests using $T_{n}$ = 271 K, which means that the bottom temperature became the warmest. This inversion can be easily seen in Figure 4b following the grey dotted line, which represents the $T_{bottom}$: before nucleation, it can be seen at the bottom of the profiles (lowest temperature), while after VISF, it moves to the top of the temperature profile. This inversion is expected because VISF is based on the fast cooling of the surface layer of the solution caused by the solvent’s evaporation when the vacuum is applied. For the ON-shelf tests at $T_{n}$ = 271 K this was observed. For all the other runs, however, whether this happens or not was unclear for several reasons. For the experiments at $T_{n}$ = 263 K, the solution was already at a temperature close to its spontaneous nucleation limit. Therefore, nucleation probably occurred before any significant gradient could develop and be detected by the IR camera.

For the OFF-shelf vials, the $T_{top}$ was already the lowest temperature before VISF, and no inversion in the profile was observed. After nucleation, however, the $T_{bottom}$ became the lowest temperature. Nonetheless, the $T_{top}$ often remained particularly low, even if not the lowest. The temperature profile along the vial followed an almost-parabolic profile, moving from the bottom to the centre, where the maximum was located. The vial centre refers to the middle point between the bottom and the cake top. Then, decreasing, moving from the centre to the top of the vial. This effect was independent of the nucleation method used. From this information, it seems that the top and bottom layers of the product were the coldest ones. In contrast, the centre of the product was warmer during the freezing of the suspended vials, independently of the application of VISF. This observation was somewhat expected considering the different heat transfer mechanisms occurring for the different loading configurations. In ON-shelf vials, heat is transferred mainly through direct contact with the shelf and conduction through the gas trapped in the gap between the shelf and the curved bottom of the vial [54]. Natural convection and radiation together with the vial walls account only for a small fraction of the total heat transfer coefficient of the system. They can be considered negligible in the first approximation. Radial and azimuthal effects are usually and reasonably neglected. However, in the OFF-shelf vials, natural convection and radiation are the only heat transfer mechanisms involved and, therefore, cannot be ignored anymore. In this situation, heat is transferred almost equally from the side and bottom of the vials and, therefore, radial effects become relevant. In this scenario, it is reasonable to assume that freezing would proceed at the same rate together with the vertical and the radial directions creating a frozen shell growing towards the centre of the vial. Unfortunately, if this is the case, the solution in contact with the vial wall would freeze first, and no advancement of the freezing front would be visible with an IR camera. The IR camera only “sees” what happens outside the vial. It depends a lot on the solution being in equilibrium with the vial wall and representing the product. That is valid for the ON-shelf vials that have negligible azimuthal and radial gradients [55]. However, the IR camera cannot give accurate information about the inside of the product cake if there are radial gradients of any kind in the solution. It would be reasonable to have an almost homogeneous temperature profile with the two minima from the outside of the vial. One minimum point at the bottom (due to natural convection) and one at the top (with the glass above the product acting as a “thermal fin”).

With the sensor’s resolution, 8 pixels were enough to cover the cake depth of 10 mm. However, this resolution did not seem sufficient to accurately measure the fast cooling of the solution surface while the vacuum was applied. Additionally, under the tested conditions, the IR sensor monitors the temperature of the vial’s external wall, which is in equilibrium with the product inside. Calculations and corrections are made to account for the temperature gradient between the outer wall and the product [32], but this affects the ability to adequately account for this fast cooling of the product surface during VISF since it assumes thermal equilibrium between the product and the vial wall.

Finally, the acquisition rate of 0.1 frames per second (fps), the highest rate available with the current sensor, leaves room for improvement. The nucleation phenomenon happens on a time scale of milliseconds, while freezing takes several minutes. Thus, the acquisition rate was not enough to capture the nucleation phenomenon accurately and with the deserved precision. However, it seemed suitable for freezing, although faster rates in the future may grant better insights.

### 4.2. Freezing Front Temperature and Position

Heat is removed from the bottom of the vial using the ON-shelf configuration. At the same time, heat is supplied to the vial side by air conduction and convection. Thus, creating a relatively large temperature gradient within the solution. In the case of the suspended-vial configuration, heat is supplied by radiation from the temperature-controlled surfaces, gas conduction, and convection. The contribution of gas conduction has been estimated to be around 90%, whereas radiation accounted for 5% and shelf/vial contact only accounted for 4%. In the case of suspended-vial configuration, gas conduction and convection accounted for 60 to 75% using a similar clearance to the one used in this study; the other contributions found for OFF-shelf vials were related to radiation, which is a clearance similar to the one used in our studies [56].

A small fraction of the water in solution forms the first nuclei during nucleation, this state of solution is called “slush”. In the range of $T_{n}$ values investigated, approximately only 3% of the bulk freezable liquid solidifies at 271 K and nearly 14% at 263 K [5]. Then, the progression of freezing depends on the cooling rate. A slight temperature jump may be observed after one of the solutes becomes supersaturated and releases latent heat of crystallisation [15]. In many cases, freezing is assumed to be complete when the glass transition temperature (for amorphous solutes) or the eutectic point (for crystalline solutes) is reached [47,57]. However, the solution may continue its freezing process at even lower temperatures [15]. Thus, the determination of the end of freezing is subject to uncertainty and debate.

**Figure A4:**
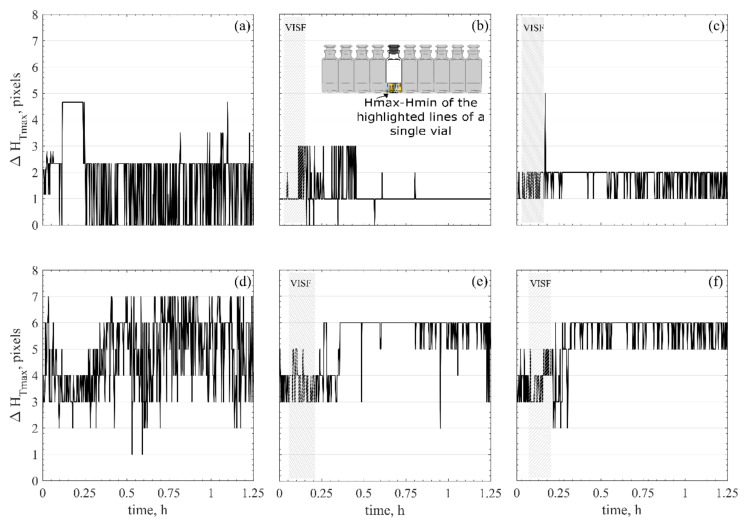
Range of measured $H_{max}$ profile values for a single vial for sucrose 5%. Top graphs (**a**–**c**) are ON-shelf, while bottom graphs (**d**–**f**) are OFF-shelf. (**a**,**d**) Spontaneous nucleation, (**b**,**e**) VISF 271 K, (**c**,**f**) VISF 263 K.

As mentioned above, the freezing interval observed in detail with the IR sensor is shorter than the required one to achieve the product’s glass transition or eutectic point. In fact, intervals between 20–45 min after ice nucleation were the ones being investigated. The temperature profiles obtained in these time intervals allow tracking the position of the maximum axial temperature $(H_{max}$) of the ON-shelf experiments (Figure 5a–c). The $H_{max}\;$position assumed to be equivalent to the freezing front position, seemed to show an apparent upwards movement, which was consistent with the expected motion of the freezing front. On the other hand, suspended vials tended to have their maximum temperature positioned around the middle of the product height (Figure 5d–f). This $H_{max}$ position indicates that heat was being removed from the bottom and top of the vial faster than from the sides during freezing, i.e., the vial walls. This observation could result from the side-wall radiation or radiation from the top and bottom surfaces, which are in contact with the bulk of the liquid. In any case, this heat removal on the bottom and top seemed to prevent observing an upwards profile of the $T_{\max}$ and, thus, the $T_{\max}$ could not be used to infer the position of the freezing front. To better understand the variability of the H profiles measured, the range of $H_{max}\;$values observed for a single vial is shown in Figure A4.

An important point from these experimental observations is the freezing front behaviour. If indeed the approximation $H_{max}\;profile≅freezing\;front\;profile$ is adequate for the ON-shelf vials; the progression of this front may be a bit different than previously modelled. In Figure 5, the $H_{max}\;$profiles of the ON-shelf vials (Figure 5a–c) linearly advanced from bottom to top in the first half of the ascending interval, i.e., at a constant progression rate. A deceleration was observed on the second half of this profile progression, and the freezing front seemed to move much slower. This deceleration could be attributed to an increment in the heat transfer resistance as the frozen layer increases. The temperature of the shelf and the temperature of the freezing front remained almost constant during freezing (excluding cryo-concentration effects). However, as freezing advanced, the thickness of the ice layer increased, decreasing the thermal gradient in the ice, which is the system’s heat transfer driving force. Ice has a higher thermal conductivity than water, with 2.14 W/mK at 273.15 K and 2.3 W/mK at 263.15 K while water has 0.6 W/mK at 293.15 K [58]. On the other hand, as the solution freezes, ice is physically removed from the liquid, leading to the cryoconcentration of the solution. For sucrose-based solutions, the conductivity decreases when the sucrose concentration increases [59], with the effective thermal conductivity at 263 K being as low as ~0.45 W/mK for 31.3% sucrose, for example. It is important to note that what is being called the “frozen layer” in this study has more ice particles formed than the initial ice-water slush produced by nucleation. However, this matrix is not completely solidified, so other factors that could pose some resistance to heat transfer may be in place. One possibility could be that the heterogeneous nature of the slush matrix could be adding increased resistance points to thermal flow. Alternatively, more likely, more heat transfer evens are happening between the shelf and the freezing front since the slush is gradually freezing, increasing the frozen layer between the vial bottom and top. In any case, these new observations and insights regarding the freezing front behaviour for the ON-shelf vials may be further analysed together with unidimensional freezing models [22,27,28,51] for a better application based on experimental data.

One-dimensional freezing models proposed by Nakagawa et al. [27] and by Arsiccio et al. [28] require the freezing rate and the temperature gradient of the frozen layer to estimate the dimension of the ice crystals formed. To use experimental data instead of simulated or assumed ones, the $T_{max}$ profile tracking is assumed as descriptive of the freezing front, both for spontaneous nucleation and VISF tests. For the ON-shelf vials, that assumption seems adequate, thus, the difference between the $T_{bottom}$ and $T_{max}$ can give the temperature gradient of the frozen layer, while the $H_{max}$ profile evolution can give the freezing rate.

### 4.3. Resulting Product Cake Structures

**Figure A5:**
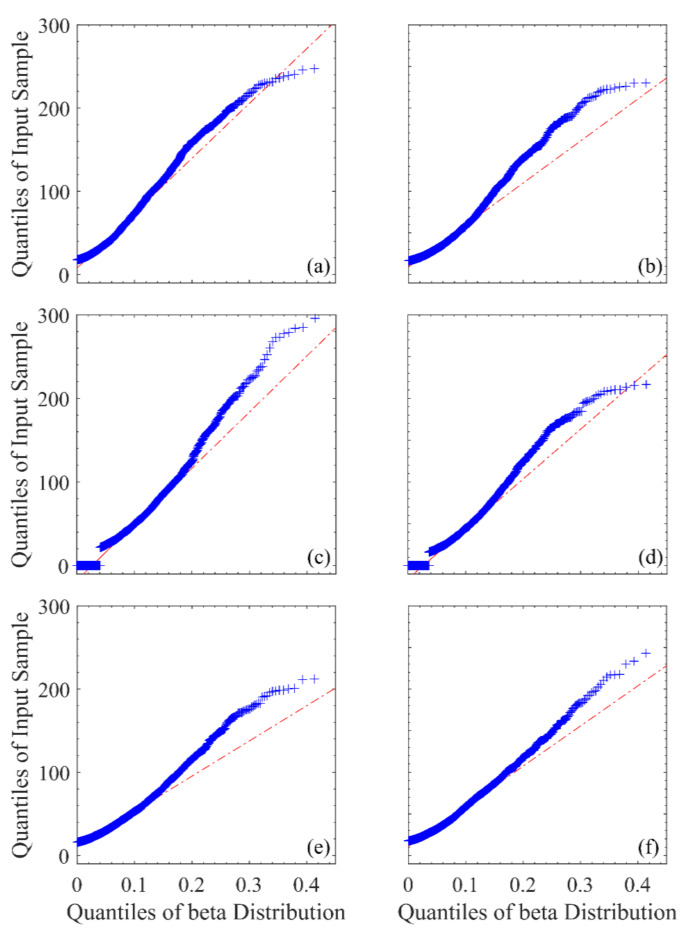
Q-Q plot for the resulting pore size distributions for each VISF test at 271 K considering a Beta distribution with α = 1.2 and β = 15. Tests on the left (**a**,**c**,**e**) are ON-shelf, while tests on the right (**b**,**d**,**f**) are OFF-shelf. (**a**,**b**) are for sucrose 5%, c and d are for mannitol 5% and e and f are for dextran 10%.

**Figure A6:**
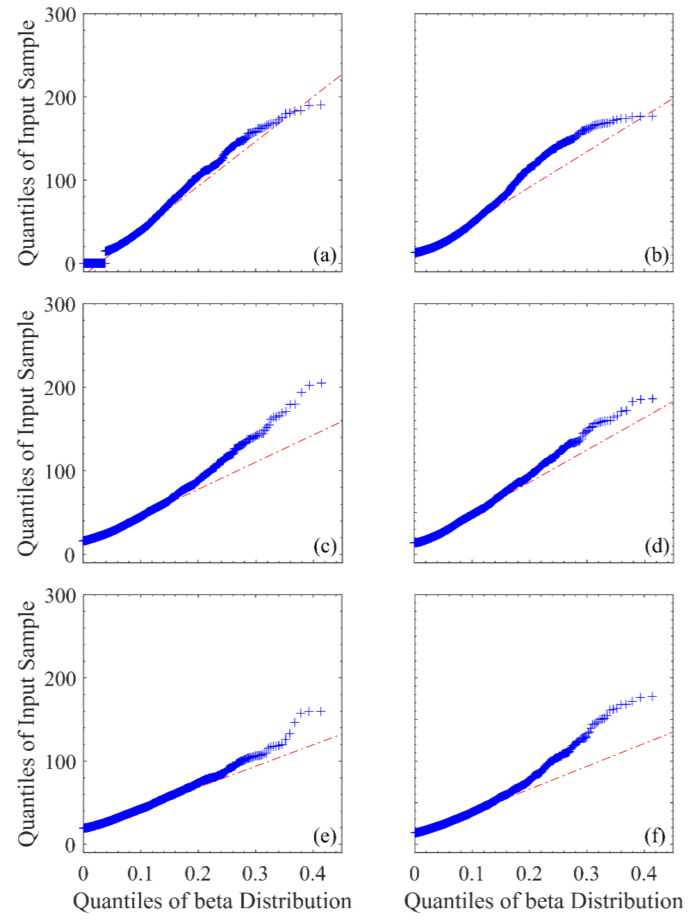
Q-Q plot for the resulting pore size distributions for each VISF test at 263 K considering a Beta distribution with α = 1.2 and β = 15. Tests on the left (**a**,**c**,**e**) are ON-shelf while tests on the right (**b**,**d**,**f**) are OFF-shelf. (**a**,**b**) are for sucrose 5%, (**c**,**d**) are for mannitol 5% and (**e**,**f**) are for dextran 10%.

The morphology of ice crystals formed during freezing strongly depends on the nucleation temperature and the cooling rate [60]. Based on the temperature gradients observed in Figure 4, more homogeneous cake structures would be expected for the OFF-shelf vials because they presented the smaller temperature gradients. Regarding the residual moisture content, a more homogeneous distribution of the moisture content using the suspended configuration compared to ON-shelf vials was previously reported [56]. Upon visual inspection of the SEM images of the resulting cake, it is hard to see any difference whatsoever. Both cakes seem very similar, and the high variability of the pore size makes any comparison difficult. Thus, an automated image segmentation software extracted the pore size distribution from the SEM images. This data extraction resulted in a couple of thousand pores extracted per sample. Using a Q-Q plot, their size distribution was tested against a beta distribution, as Colucci et al. [29] proposed. These results are displayed in Figure A5 and Figure A6 in the Appendix A.

Although the pore size distribution data is very spread, the variance and the interquartile range consistently showed lower values for the OFF-shelf runs than the ON-shelf ones. These results indicate that the use of a suspended configuration does improve product homogeneity. However, the loading configuration had a modest effect. The nucleation temperature was the major player in determining the resulting ice crystal size. When VISF was applied, although the top surface of the liquid being frozen cooled down, the remaining solution was at a uniform temperature. This more uniform temperature may be the reason behind the improvement in the cake uniformity observed for samples produced by VISF. When the shelf ramped freezing is used and uncontrolled freezing is applied, the vials directly in contact with the shelf undergo a relatively fast-cooling ramp. Under these circumstances, the bottom of the vial tended to be colder than the rest of the solution. When nucleation took place, different layers of the solution could have nucleated at different temperatures. During nucleation, a temperature gradient could result in a less homogeneous cake, with smaller ice crystals (and consequent dried cake pores) in the bottom and larger crystals at the top. Fang et al. [61] found that vials that nucleated at the same temperature (261 K) but had different freezing rates after nucleation also presented different resulting pore sizes in the resulting cake. The slowest freezing rate used (0.1 K/min) resulted in larger pores (28–60 µm) while the fastest freezing rate (2.5 K/min) resulted in smaller pores (22–48 µm) [61]. According to these findings, the nucleation rate plays a role in the resulting ice crystal size and the freezing rate during freezing. In this case, some differences should be observed between the OFF-shelf and the ON-shelf vials. The OFF-shelf vials would have a slower freezing rate and, by this logic, should have a resulting cake structure with slightly larger pores. This correlation could not be observed in detail, however. From the experimental data obtained, the effect on the freezing rate on the pore size distribution seemed to be negligible. The nucleation temperature was the only parameter that seemed to strongly affect the pore size distribution.

## 5. Conclusions

An IR camera was used to track the freezing dynamics of pharmaceutical solutions in vials in the case of two different loading configurations. A deeper insight into the freezing phenomena was obtained, and many notions were confirmed or deduced.

As previously published, VISF works very well to induce nucleation in a short time interval (~100 s) and with a good intra- and inter- vial uniformity. The suspended (OFF-shelf) vials have a narrower overall temperature profile gradient than the ON-shelf ones due to their different heat transfer mechanisms.

The IR camera works very well to monitor the VISF tests since the batch is more homogenous than when using spontaneous nucleation. The profiles become clearer to be observed compared to this tool applied to spontaneous nucleation batches. However, the presented IR tool is unsuitable for high-speed events and when the thermal gradients are not pronounced enough. Thus, higher thermal resolution in terms of pixels and faster data acquisition rates are required to observe the nucleation phenomenon adequately.

The pore size distribution of cakes could be described by a beta distribution as proposed by Colucci et al. [29]. Furthermore, using the suspended vial configuration applied to VISF, very homogeneous cakes can be obtained.

Assuming the $T_{max}$ during the freezing interval to be the freezing front temperature and acquiring its axial position over time, a direct, empirical approach for the one-dimensional models from Nakagawa et al. [27] and Arsiccio et al. [28] could be used. This experimental approach using the IR camera could support the in-line optimisation for the primary and secondary drying steps based entirely on empirical data collected during the freezing stage. Moreover, according to the models and assumptions previously used by Nakagawa et al. [27] and Arsiccio et al. [28], the freezing front followed a linear progression. However, from the experimental observations, if the tracking of the $T_{max}$ can be adequately assumed to represent the freezing front for the ON-shelf vials, adjustments to the models or even new models could be proposed to predict the ice crystal size. Future work will therefore focus on the application (and the applicability) of the aforementioned models to predict the pore size distributions using exclusively data obtained by direct observation of the freezing step using an IR camera.

## Figures and Tables

**Table 1:** Changes in the temperature gradient observed for the various tests carried out in this work.

Type	Solution	Nucleation	$T_{bottom}\;\mathrm{before}\;\mathrm{Nucleation}$	$T_{bottom}\mathrm{after}\;\mathrm{Nucleation}$	**VISF Inversion Observed?**
ON shelf	sucrose 5%	Spontaneous	Lowest	Lowest	–
	sucrose 5%	VISF 271 K	Lowest	Lowest	Yes
	mannitol 5%		Lowest	Lowest	Yes
	dextran 10%		Lowest	Lowest	Yes
	sucrose 5%	VISF 263 K	Lowest	Lowest	No
	mannitol 5%		Lowest	Lowest	No
	dextran 10%		Lowest	Lowest	No
OFF shelf	sucrose 5%	Spontaneous	Highest	Lowest	–
	sucrose 5%	VISF 271 K	Highest	Lowest	Unclear
	mannitol 5%		Highest	*	Unclear
	dextran 10%		Highest	Lowest	Unclear
	sucrose 5%	VISF 263 K	Highest	Lowest	Unclear
	mannitol 5%		Highest	Lowest	Unclear
	dextran 10%		Highest	*	Unclear

* gradient not wide enough with $T_{bottom}$ not necessarily at the highest or lowest position.

## Data Availability

Not applicable.
